# The Nursing Supplement

**Published:** 1888-04-14

**Authors:** 


### The Nursing Mirror.

### En Passant.

INTRODUCTORY.—We wish to call the attention of our readers to the different branches of this special supplement. We have attempted to allot the space so as to secure the largest amount of information in the most concise form, and to give to every branch of the nursing profession the notice and attention it merits. Every effort will be made to secure the latest information from all hospitals and institutions, both at home and abroad. To obtain this we seek the friendly co-operation of all matrons and nurses in making known to us the various vacancies and appointments as they occur, and we also invite correspondence on all subjects connected with nursing, promising to every letter our best attention. At the request of many of our readers an Exchange Column has been started; also a column for Notes and Queries. All this information is issued entirely as a free supplement to THE HOSPITAL, so that a nurse who takes in this paper secures not only all the nursing news, but the stories, articles, and general reading provided in the entire paper.

ESCAPADE OF A FEVER PATIENT.—A young man named Alfred Hartley was discovered one morning last week by a policeman wandering about the streets of Bradford, nude, with the exception of a shirt. He was arrested, and inquiries showed that he had escaped during the night from the Borough Fever Hospital in a fit of delirium from typhoid fever. He had overpowered his nurse, locked her in her room, and then got through the window. Except for severe cuts on his feet he seemed little the worse.

A WORD OF WARNING.—A young lady medical student has lately died in Paris from blood poisoning. The virus was inadvertently introduced into the system through a slight abrasion on the chin; and the sad fate which followed is one more warning to nurses to be excessively careful in covering up all cuts and scratches. It is a warning which is often given, and more often unheeded. Every matron of experience can remember nurses under her care who have suffered through inattention to this well-known precaution.

A BABIES' HOSPITAL.—We quote the following from a New York paper:—"The ladies of the Cribside Committee of the Babies' Hospital will give a tea at the hospital building, 459, Lexington-avenue, next Saturday afternoon, for the purpose of raising a fund for the endowment of a bed at that institution. This hospital has just purchased a house on Lexington-avenue, and is now ready for active work. Out of the 10,000 beds in the New York hospitals, only 27 are devoted exclusively to children under one year of age. The Babies' Hospital is simply an enlargement of this work, and its wards will be under the constant care of nurses from the Nightingale Training School. Donations of children's clothing, blankets, flannels, old linen, &c., for hospital uses will be gratefully received, and a special request is extended to young girls to lend their needle services."

HOUSES OF REST.—Miss Hicks, the new matron of the Hospital for Sick Children, Great Ormond Street, is one of those who have been in the habit of getting personal friends to take nurses into their houses for a period of rest. Many nurses who have thus secured change of air and all the sweet comforts of home life, speak very highly of the scheme. The proposal to build a holiday house, or sort of convalescent home, for nurses, is not nearly so popular. To begin with, nurses have enough of institutions while they are at work, and what they seek is thorough change, such as a private house alone can offer them. They want to get away altogether from the subject of nursing; not to be thrown into company with more nurses, who will be certain to talk hospital gossip from morning till night. Those ladies who receive a nurse as a visitor into their pretty country homes, and make much of her during her short fortnight holiday, confer on that nurse the most complete and healthy change which it is possible for her to have. Unluckily, only a few ladies are willing to undertake this work, and therefore only a few nurses can obtain the benefit.

"A HAPPY DAY?"—In a copy of the *St. James's Gazette* last week there was an article entitled "A Nurse's Day in Hospital," which we hope greatly exaggerated many of the details. To begin with, the writer states that most hospitals draw their staff of nurses from some Anglican sisterhood, a fact which a glance at Whitaker or any other source of information will prove to be false. Three or four of our metropolitan hospitals are nursed by sisterhoods, but by far the largest number have their own private staff. Then the writer complains of having to rise at half-past five a.m. to attend early service, which is, however, no part of her nursing duties, and should not be included in an account of a day's work in a hospital. The most remarkable statement of all is that only a quarter of an hour is allowed for dinner, and that though the food is good, the nurses often have not time to eat it. We have constantly heard complaints of the food provided in nursing institutions, but never before heard of nurses hurried away from a pleasant meal. In the hospital where the writer was trained hurry seems to have been the rule, and we cannot show the drift of the article better than by quoting the account of Saturday afternoon: "This is the time when operations are performed. The work goes on from one p.m. to six p.m., while patient after patient is chloroformed and laid upon the table. The nurses wash out sponges and stand ready to hand instruments. The work is critical, the surgeons brusque and excited, and the evening finds every one exhausted." The details told of the arrangements, such as the half-hour for the probationers' tea, when a servant waits on them; the time of evening service, &c., leave no doubt as to which hospital is meant to be described, and, unless we are much mistaken, the article will provoke a reply from those in authority, unless they consider the statements of a probationer beneath their notice. What we specially admire is the account of how patient after patient is laid upon the table. Evidently there is a ceaseless stream of those requiring operations, all saved up for Saturday afternoon.

*A new and large field for nursing* will be opened next week at the new buildings of the Great Northern Central Hospital, in the Holloway Road. Hitherto the Great Northern Central has laboured under serious disadvantages from a nurse's point of view. The old building was very small, containing only 33 beds; it was eminently unsuitable for the purposes of a hospital, being merely a block of badly-adapted private houses; it was placed in a thickly-populated and by no means agreeable neighbourhood, and its internal arrangements for the nurses' comfort, though the best that could be secured under the circumstances, were by no means delightful. All these disadvantages will come to an end next week. The new building, a part of which will then be opened, will be one of the best placed, best arranged and healthiest hospitals in London. It stands on very high ground close to Highgate. When finished it will give accommodation to nearly one hundred and fifty patients. There has never been any difficulty in obtaining nurses at the old hospital, in spite of its manifest drawbacks as a nurse-training school. In the future it is probable that the high and healthy situation of the building and other improved circumstances will cause its nursing appointments to be specially sought after.

### The Registration of Trained Nurses.

The Joint Committee of The Hospitals Association appointed to consider the question of a system of registration for qualified nurses have unanimously agreed to the following important report. It proves that many of the statements made of late on this subject were premature. No system of central registration will probably be acceptable or successful for some years to come, if at all. The report is as follows :—

Your Committee considered that the first step towards a right knowledge of the subject was to make a formal inquiry into the practice and wishes of the various nurse-training schools in London and throughout the country, with the view of eliciting whether they were desirous of establishing a collective Register; and, if so, to state the length of the curriculum, the syllabus of education, and the character of the certificate which they deemed necessary to qualify a nurse before her name should be entered on the proposed Register. To obtain the fullest information on these and other incidental points connected with nurse training, the Committee put itself in communication with thirty-four establishments in England and Scotland which profess to educate nurses. Of this number, twenty-four were associated with Medical Schools, seven were ordinary Medical and Surgical Hospitals, and three were Union Infirmaries. Eight of the hospitals communicated with have taken no notice of the application; one curtly refused to have anything to do with it; six acknowledged receipt, with a promise that the document should receive consideration; nineteen, comprising seventeen large Hospitals and two Union Infirmaries, have contributed more or less information. The questions were addressed through the Secretaries to the Chairmen of the House or Nursing Committees of the respective hospitals ; and it may be fairly assumed, since some of the questions relate to matters outside domestic management, that difficulties and delays in answering them have arisen, from the desire, on the part of the committees, to consult the superintendents of nurses and the medical authorities of the hospitals on the subject. Some of the answers are simply the individual opinions of the matrons, or of medical superintendents, or of some prominent member of the medical staff of the hospital and may, or not, be those of the committees of management, who, as a rule, are unwilling to pledge themselves to any special course of action. The doubtful character of many of the replies to the questions, and the entire absence of any reply at all to others, indicate considerable divergence of opinion among the authorities consulted, and all tend materially to increase the difficulties surrounding the subject.

Question I. Do you consider a general system of registration desirable for qualified nurses ? may be viewed as a key to the whole series, for it would naturally follow that in the case of a direct negative there would be no occasion to enter any reply to the others. Rather less than one-half of the replies are in the affirmative, while the bare majority, comprising, however, the leading and best known training-schools, desire to be let alone. The grounds of the latter for this opinion are based on the facts that each has established a Register of its own to meet its special requirements; that they would lose rather than gain by any system partaking of amalgamation; and that the time has not arrived for a central organization to be delegated with authority to control the action of the various bodies, commercial and charitable, entrusted with the management of nurses. There is every reason to believe that the following training-schools concur in these opinions : The Nightingale Fund Committee associated with St. Thomas's Hospital and other institutions, the London Hospital, Guy's Hospital, St. George's Hospital, Westminster Hospital, King's College Hospital, together with the Royal Infirmaries of Manchester, Liverpool, Glasgow, and Edinburgh. It is highly probable also that many of the hospitals which have not yet answered the questions addressed to them have refrained from doing so from an unwillingness to entertain suggestions which would interfere with their local organisation.

Questions II. and III. have reference to the central agency through which registration ought to be organised, should the answer to Question I. be in the affirmative. Five of the institutions declare for the General Medical Council for Education and Registration, four for a separate organisation associated with the training schools, and two with The Hospitals Association. Failing the Medical Council undertaking the duty, two of the above would prefer The Hospitals Association, two a separate organisation, and one the British Nurses Association if it was properly constituted.

Question IV. refers to the length of the curriculum considered desirable for a nurse to undergo prior to her having her certificate, or her name entered on the proposed Register. Here opinions vary very much. Nine think that a nurse should serve three years before being certified, four consider two years sufficient, three suggest that each should serve one year's probation and one year as nurse, and one only would limit the time to one year.

Questions V. and VI. relate to the instruction given to the nurse-pupils during their term of probation. Oral instruction by means of lectures and demonstrations appears to be carried out in all the institutions which have responded, with the exception of two, and considerable stress in two instances is laid on the expediency of the tuition being followed up by practical demonstrations from a female instructor. The nurse's knowledge on the subject of lectures is tested in about one-half of the replies to this question ; but the remark is appended in more than one case, that it frequently happens that those who distinguish themselves most by answering questions turn out to be the least efficient nurses. With regard to the amount of instruction, opinions vary, but the majority consider that lectures on medical or surgical subjects should be given once a week, apart from the practical instruction the probationer receives from the head nurse or sister of the ward.

Question VII. alludes to the terms employed in the nurses' certificates, with the view of obtaining greater harmony, and, if possible, uniformity. At the present time considerable diversity exists in the wording of the certificates, and several of the provincial hospitals consulted are ready to alter the terms used should a uniform standard be adopted. In a very few, the length of service only is stated ; others append to the period a commendation to the effect that the nurse has performed her duties "to the satisfaction," or "to the entire satisfaction" of her employers, while the majority express approval by varying terms ranging from "highly satisfactory" to "tolerably good." In a few instances space is left for any remarks it may be thought necessary to add. The Nightingale Fund Committee have not hitherto issued certificates to their registered nurses, but are contemplating the expediency of doing so.

As to Question VIII., which refers to the persons authorised to sign the certificates, there is a general agreement ; for, with the exception of the Edinburgh and Glasgow hospitals, where the medical superintendents sign in the name of the governing bodies, all the others append three signatures, comprising that of the matron, a representative of the medical officers, and the Chairman of Committee. In most provincial hospitals the house surgeon signs as the representative of the medical authorities, but in the London hospitals this duty is done usually by one of the medical instructors.

It is manifest to your Committee that the obstacles in the way of organising a general system of registration increase with the knowledge possessed of the working of the various institutions in which nurse-training is carried on. It is equally evident that, unless there is a general unanimity among the training-schools as to the basis on which such a system can be inaugurated, any attempt to form a voluntary Register must necessarily prove a failure, while, in the present progressive condition of the nursing question, legislative interference would be attended with disastrous results. The fact that most of the large hospitals, some of which have long been known as pioneers in the movement for the better education of nurses, disapprove of an innovation which they honestly believe would in a manner dissociate the nurse from her parent school, practically settles the question. A central organisation, possessing a separate jurisdiction, cannot fail more or less to affect the individuality, the healthy rivalry, and the *esprit de corps* which characterise the members of the best training-schools, by reducing them to a dead level with others, which are far from possessing the same advantages. It is to the manifest benefit of every hospital to be able to adapt its training, certificating, and registering, to meet its own local requirements, and the nature of the duties the nurse may afterwards be called on to perform when she leaves the service of the hospital.

If your Committee's inquiries have not been fortunate enough to elicit a consensus of opinion with regard to collective registration, they have been eminently useful in showing the necessity of every hospital possessing a Register of its own, formulated on a principle of easy reference comprising the length of the curriculum, with the individual attainments of each of its nurses. The certificates awarded, it is needless to say, should be verbatim copies of the terms employed in the Register, whether the curriculum has extended over one, two, three, or more years. Such appears now to be the practice in the best training-schools, and it is hard to believe that it can be surpassed by a collective registration. The results of the inquiry all tend to confirm the opinion that it would be premature on the part of the Committee to recommend to the Council a common basis on which a general system of registration for nurses should be framed, however advisable it may be for every hospital, being also a nurse-training institution, to amend and consolidate its own educational training in keeping with the improvements which of late years are well known to have been introduced into most establishments of this character.—Signed on behalf of the Joint Committee on Registration, J. C. Steele, chairman; adopted by the Council of The Hospitals Association, J. S. Bristowe, M.D., F.R.S., president.

### Lectures.

April 13th, at 15, Buckingham Street, Strand, at 7.45 p.m., R. H. Humphreys, Esq., on the Nursing of Cases of Heart Disease. Admission 6*d*.

April 13th, at the Parkes Museum of Hygiene, at 8 p.m., Sir Douglas Galton on Ventilation, Measurement of Cubic Space, etc.

April 13th and 30th, at the Memorial Hall, Farringdon Street, at 7.30 p.m., Dr. Richardson on Ideal Food. Admission 6*d*.

The course of lectures on Medical Nursing at the London Hospital is this year being delivered by Dr. Anderson, instead of by Dr. Sansom as formerly.

A course of five lectures on Domestic Hygiene, given by Dr. Schofield at the Parkes Museum, has just drawn to a successful close. The closing lecture was entitled "Home Nursing," and two trained nurses were in attendance to give practical illustrations of the doctor's directions. This last lecture certainly seemed the most popular of the course, perhaps because of the presence of the nurses in uniform. There is to be an examination shortly, and the Duchess of Albany will present certificates to those who pass successfully, on May 5th, at the Museum.

### Fresh Fields.

### NURSES FOR THE EGYPTIANS.

On the 15th of February Sir Sydney H. Waterlow, Bart., treasurer of St. Bartholomew's Hospital, who, as we intimated last week, has been travelling in Egypt, had a private interview with the Khedive. He was introduced by the British Minister, Sir Evelyn Baring.

Sir Sydney submitted to His Highness a proposal for the establishment of a nurses' home attached to the Kasr-el-Aini Egyptian Hospital in Cairo, and the introduction of a few trained English nurses to control and educate the native female nurses in the performance of their duties.

The Khedive was much interested in the subject, and after a careful discussion of the question, gave his general consent to the scheme on two conditions.

First, that the English nurses should never attempt to proselytize the native patients.

Second, that no female nurse should attend upon a male patient. These conditions were immediately accepted, and the interview terminated.

The scheme has since been formulated in writing, the necessary money for this year has been promised by the Minister of the Department controlling the hospital, and is to be taken from the reserve fund. If the proposal works well a special item is to be inserted in the Budget for 1889 to meet the annual cost of the home.

The European population of the City of Cairo are delighted at the prospect of having some trained English nurses available on proper payment, in the event of any case of serious illness.

The scheme includes an arrangement for providing such extra number of trained nurses as experience may render necessary.

Early this month the proposal was formally submitted to the Khedive and the General Council of Ministers, and officially approved. It will come into operation at once, as the nurses are to be sent out by Sir Sydney immediately he returns to London early in April, and they are expected to be on duty by May 1st.

Memorandum of the Scheme for Establishing a Nurses' Home at the Kaisr-el-Aini Hospital at Cairo.

February 21st, 1888.

The Institution although independent of the Egyptian Government, would undertake in return for a subsidy the supervision and training of the Nursing Staff of the Female Wards of the Kaisr-el-Aini Hospital.

It would in the first place consist of two nurses of equal rank and standing, each receiving board, lodging, attendance, uniform, and £84 per annum. £25 would be paid to each for the passage out from London, and a similar sum for passage home should her services no longer be required before the expiration of the first year.

The Institution would depend for its funds—

Firstly, on the Government subsidy.

Secondly, on the earnings of the nurses outside the Hospital.

Thirdly, if necessary, on voluntary subscriptions.

At the commencement the whole management of the Home would be vested in the Senior Medical Officer of Kasr-el-Aini (Dr. Milton) with a proviso that should the number of nurses so increase as to render it advisable a Board of Trustees should be elected from the subscribers to the Fund.

An annual subscription of £2 would constitute a Governor. A donation of £10 a Life Governor.

The work of the Nurses' Home would be:

First, governmental, viz., the supervision and control of the nursing of the Hareem or Female Wards of Kasr-el-Ain Hospital.

Second, private, viz., the nursing in their own houses of such Europeans or natives as are willing to pay the charge to be determined later on.

In drawing up the regulations for the management of the Home, care will be taken that the working of the Hospital will at no time be interrupted, and that only such portion of the nurses' staff shall be withdrawn from their governmental duties as can be spared from time to time.

In carrying out this scheme, the first necessity is to obtain the Government subsidy, and with this view application has been successfully made to the Board of Health for the sum of £300 per annum.

This sum has been fixed so as to cover all expenses for two nurses on the following estimate:

| | |
|---|---|
| Pay for 2 nurses at £7 per month ... ... | £168 |
| Board ,, 3 ,, ,, ... ... | 72 |
| Extra furniture, uniforms, &c. ... ... ... | 60 |
| | £300 |

As it will be impossible for the nurses to commence work before the middle of April or beginning of May, their travelling expenses will be more than covered by the sum allowed for January, February, and March.

Should the scheme prove to work well, it will probably at the end of 1888 be able to supply for the same subsidy, a third nurse for the Alexandrian Hospital.

### Anecdotes of Personal Adventure.

One afternoon, in a medical ward, a celebrated physician was giving a clinical lecture on a throat case. It was very hot, and most of the patients were sleeping, save the poor fellow who was being examined by relays of students, and a patient at the far end of the ward, who was very feverish and muttered constantly to himself. The sunshine streamed through the long windows, and fell in golden patches on the polished floor. The monotonous tone of the lecturer droned on, and the students lounged against the walls and yawned, and few made any pretence of listening. The sister had been standing patiently, ink-pot in hand, for over an hour, and looked very white and weary; the nurse's arm ached with holding the lamp long in one position, and the overworked house physician who was diligently taking notes, seemed to be the only person who was profiting by the discourse. Suddenly the patient at the far end of the ward called out "murder!" and, springing out of bed, came running down the ward with outstretched arms. The students, delighted with a break in the monotony of the afternoon, rushed forward to secure him, but before they reached him he had seized another patient by the throat with the evident intention of strangling him. Of course it was the work only of a few seconds to pull him off and carry him back to bed, but still the short excitement had completely aroused the ward from its semi-somnolent condition, and the thread of the lecture was broken. The disgusted physician retired with dignity, an order was procured for a male attendant, and that small adventure was over without any ill results.—*Nurse B.*

An insane artist entered his sister's room, having a large knife in his hand. "Kate," he said, "I am painting the Princess de Lamballe in the Revolution, and I want your head for a model." "To be sure, John," she replied; "you shall cut it off at once, but I am afraid you might spoil this collar that I'm so fond of. I'll just go and change it and come back to you." "Don't be long," he said; "I'll wait here for you."

### Notes and Queries.

To Correspondents.—1. Questions may be written on post-cards. 2. Advertisements in disguise are inadmissible. 3. In answering a query please quote the number. 4. If a private answer is desired, a stamped addressed envelope must be forwarded.

[The many questions with regard to the Pension Fund are held over this week, but will receive attention in our next number.

*E. B. Johnson.*—You must be provided with your own thermometer. Indeed it is best to take two with you, for they are easily broken, and in a country place are difficult to replace.

(1.) *Quotation Wanted*:—

"I, in the midst of those who suffer so,
Who needs must somewhat share the daily pain
Which each of ye, beloved, must endure,
Must also seek some comfort, and some strength
Of hope to live and suffer by."

(2) *A Bracing Hospital Wanted.*—Can you or any of your readers inform me of a hospital in a bracing and healthy situation, where a lady could have three months' training in general nursing from the middle of June to the middle of September, and what the terms would be?—G. E. D., Davos Platz.

(3) *Maternity Hospital: Without Payment.*—Would you kindly inquire, through your valuable paper, if there be any terms upon which one can enter a maternity hospital without paying.—Constant Reader, Derbyshire.

(4) *Training for Nurses.*—The secretary to a nursing institution wishes to know of hospitals where there is a good medical and surgical training to be had for nurses, either free, or at a small charge.

### Appointments.

Miss Phillippa Hicks, late assistant matron at King's College Hospital, has been appointed lady superintendent to the Hospital for Sick Children, Great Ormond Street, in place of Miss Catherine J. Wood, who has resigned. Miss Hicks is the daughter of a clergyman in Yorkshire, and was trained at St. Thomas's Hospital. She is about twenty-eight years of age, and has been engaged in nursing about eight years. She is a general favourite amongst her fellow workers, and the appointment has caused universal satisfaction.

Miss Florence Smedley has been appointed Sister Darker at St. Bartholomew's Hospital, in place of Miss Loch, who has gone to India. Miss Smedley has lately completed her training at St. Bartholomew's, having taken a gold medal at the nurses' examination last summer.

### Vacancies.

The following vacancies are announced:—

**Matrons.**

General Infirmary, Derby; £80

Lewes Infirmary.

Wellington College; £70.

Bournemouth Cottage Hospital.

House of Industry Hospitals; two; £60.

Southend Cottage Hospital.

National Orthopædic Hospital, Great Portland Street; £50.

Home for Chronic and Incurable Diseases, Leamington.

**Nurses.**

Chelsea Infirmary; £18.

Dumfries Infirmary.

City of London Hospital, Victoria Park; £20.

Burton-on-Trent Infirmary; £23.

Liverpool Cancer Hospital.

Victoria Home, Bournemouth; several.

Jersey Institution for Trained Nurses; several.

Workhouse Infirmary Association; several.

Hospital for Consumption, Ventnor; assistant; £18.

Surrey County Hospital; assistant.

Salop Infirmary; head; £25.

**District Nurses.**

Glasgow Nursing Association; several.

Ashton-under-Lyne; £40.

Sutton, Surrey; £50.

Dublin.

**Probationer.**

Liverpool Cancer Hospital.

**Midwives.**

Glasgow Nursing Association; several.

Ashton-under-Lyne; £40.

### Exchange and Mart.

Under this heading we propose to insert purely Exchange advertisements, at a charge of sixpence for eighteen words, or three insertions for one shilling. It must be distinctly understood that ordinary advertisements will not be inserted here.

The following vacancies are announced this week, the amount of salary in each case being given after the name of the institution :—

**Resident Medical Officers.**

Birkenhead Borough Hospital. Junior House Surgeon.—£50 per annum, with board, &c.
Birmingham General Dispensary. Resident Surgeon.—£150 per annum, with £30 extra for cab-hire.
Central London Ophthalmic Hospital, Gray's Inn Road. House Surgeon.
Derby Borough Asylum. Medical Superintendent.—£350 per annum, with furnished house.
Infirmary for Children, Myrtle Street, Liverpool. House Surgeon. —£85 per annum, with board.
St. Luke's Hospital. Resident Clinical Assistant.

**Non-resident Medical Officers.**

Durham Union Workhouse. Medical Officer.—£50, and extras.
Liddell Provident Dispensary, Jarrow-on-Tyne. Medical Officer. —£200 per annum.
Oughterard Union, Cloonbur Dispensary. Medical Officer. £102 per annum, and fees.
Oughterard Union, Lettermore Dispensary. Medical Officer.—£132 per annum, and fees.
Tarbat Easter Parish, Ross shire, N.B. Medical Officer.—£115 per annum.

---

It is announced that Dr. Thomas Keith, whose record of success in gynæcological operations is a matter of pride to the medical world of Scotland, purposes leaving Edinburgh for London. We congratulate the metropolis and condole with the northern city on this resolution.

---

On Thursday last the Blackheath St. Cecilia Choir, under the management of Miss Rucker, kindly gave a concert to the patients in the Seamen's Hospital at Greenwich. The programme was carefully arranged to suit so cosmopolitan an audience, and contained songs in Spanish, Russian, Norwegian, German, and Italian, besides English, Scotch, and Irish ballads. The concert was given in the recreation-room, and at the same time members of the choir sang on the different floors to those sailors who were unable to leave their beds.

---

Mr. W. H. Paling, of Sydney, New South Wales, is celebrating the centenary of the Colony in a noble manner. He has signified his intention of setting aside his estate at Camden, extending to four hundred and fifty acres, and a capital of £10,000 for the purpose of establishing and endowing a convalescent home for the benefit of the inhabitants of Sydney. Mr. Paling has stated that in order to identify the institution with the time when it was founded, he is desirous that the hospital may be named after the governor of the colony at the time of the centenary. It will therefore be called after Lord Carrington, but it is to be hoped that the name of the generous donor will also be commemorated in some way.

---

At the annual meeting of the Hull Royal Infirmary, Mr. J. A. Wade drew attention to the increasing amount wine, spirits, etc., came to in reckoning the hospital expenditure. Apparently satisfactory reasons were given for the increase last year, but we think a very complete explanation is necessary for the fact that the sum spent on this item has been steadily growing larger for five years, irrespective of the number of patients treated in the institution. The statistics of the matter are as follow : Year 1883, 2,025 inmates, expenditure on wine, etc., £90 18*s*. 2*d*. ; 1884, inmates 1,705, wine, etc., £92 17*s*. 10*d*. ; 1885, inmates 1,420 ; wine, etc., £125 17*s*. 10*d*. ; 1886, inmates 1,822, wine, etc., £154 19*s*. 11*d*. : 1887, inmates 1,873, wine, etc., £176 7*s*. While no one who cares for the welfare of hospitals would wish to see a patient who needed it stinted of alcohol or any other comfort, there is a growing feeling that the less it is used, consistently with the safety of the patient, the better it will be for his ultimate welfare. Brandy is a good thing in many cases, but it is not quite the panacea the old women used to think it.

The managers of the Walsall Hospital seem to have awakened to a sense of their duty. At a meeting of the Executive Committee the following resolution has been adopted : Resolved, "That this committee considers the honorary surgeons are responsible for the treatment of all fractures or other serious cases coming to the hospital, but where, from whatever cause, a case is first treated by the sister in charge, she shall direct the patient to see one of the honorary surgeons at the first possible opportunity ; and that a register be kept recording all such cases and on what day the patient was seen by one of the honorary surgeons, as directed by the sister in charge." That is well so far as it goes, but it does not go far enough. There seems to be more than sufficient work at the Walsall Cottage Hospital for an efficient house surgeon, and the opportunities for gathering experience are apparently many. A house surgeon should be appointed without delay, and if the income will not allow of such an addition to the expenditure, a grant should be made from the poor rates

---

The Rev. Fitzhenry William Ellis, chaplain of St. Mary's Hospital, Paddington, died on the 28th ult., at his residence, 21, Gloucester Place, Hyde Park. The deceased graduated at Trinity College, Cambridge, in 1840, and was ordained priest by the Bishop of London in 1844. In the following year he was appointed chaplain on the staff of the Bengal Ecclesiastical Establishment, and was present at the siege and capture of Delhi, for which he received medal and clasps. In 1865 he was chaplain of the field forces at the storming of Dewangiri Bhootan, and again received a like honour. Mr. Ellis retired from the service in 1872, and was elected chaplain of St. Mary's Hospital in 1874. During his long service at the hospital he won the esteem and affection of his fellow-workers ; he managed the Samaritan Fund entirely himself ; and on the occasion of the sudden death in 1881 of Mr. Wilkinson, the then secretary, Mr. Ellis stepped in and performed for some months the whole of the secretarial duties, in recognition of which he was unanimously elected an honorary life governor of the Hospital.

---

A most absurd and illiberal proposal has been brought before the trustees of the Bristol Royal Infirmary, namely, that no one shall be appointed to any honorary staff appointment in the infirmary, whatever other qualifications he has, unless he possesses also a diploma from one of the London medical colleges. The absurdity of such a regulation will be patent to all who know the comparative values of diplomas, but it may easily be so put before the eyes of laymen as to induce them to do a great wrong to many skilful practitioners, and to offer a great insult to colleges whose diplomas no one can obtain without sufficient knowledge of the theory and practice of medicine. It is true that such a regulation already exists in many hospitals, but that is a matter for regret and reform rather than imitation. We call to mind in this connexion the experience of a young friend of ours, who, having passed with distinction the examinations for the M.B. and C.M. of Edinburgh, was refused the house surgeoncy of an English provincial hospital because he did not possess "a London degree." Being desirous, for reasons not unconnected with the heart, of remaining in the neighbourhood, he came to London and fitted himself for the post to which he aspired by taking the diploma of L.S.A.—a qualification which perfectly satisfied the *exigeant* hospital governors. We are quite sure that the authorities of the English Colleges of Physicians and Surgeons have no sympathy with this desire to create an unjust monopoly for them ; and with regard to the Bristol folks we can only say that, as they do not propose that their new rule shall come into force before 1891, we shall cherish the hope that before then they will see the wisdom of rescinding it.

